# Advanced Engineering Strategies for Periodontal Complex Regeneration

**DOI:** 10.3390/ma9010057

**Published:** 2016-01-18

**Authors:** Chan Ho Park, Kyoung-Hwa Kim, Yong-Moo Lee, Yang-Jo Seol

**Affiliations:** 1 Department of Nanobiomedical Science & BK21 PLUS NBM Global Research Center for Regenerative Medicine, Dankook University, Cheonan 330-714, Korea; chanho@dankook.ac.kr; 2 Department of Periodontology and Dental Research Institute, School of Dentistry, Seoul National University, Seoul 110-749, Korea; khk6911@gmail.com (K.-H.K.); ymlee@snu.ac.kr (Y.-M.L.)

**Keywords:** periodontal tissue complex, multiple tissue formation, cell sheet engineering, 3-D printing technology

## Abstract

The regeneration and integration of multiple tissue types is critical for efforts to restore the function of musculoskeletal complex. In particular, the neogenesis of periodontal constructs for systematic tooth-supporting functions is a current challenge due to micron-scaled tissue compartmentalization, oblique/perpendicular orientations of fibrous connective tissues to the tooth root surface and the orchestration of multiple regenerated tissues. Although there have been various biological and biochemical achievements, periodontal tissue regeneration remains limited and unpredictable. The purpose of this paper is to discuss current advanced engineering approaches for periodontal complex formations; computer-designed, customized scaffolding architectures; cell sheet technology-based multi-phasic approaches; and patient-specific constructs using bioresorbable polymeric material and 3-D printing technology for clinical application. The review covers various advanced technologies for periodontal complex regeneration and state-of-the-art therapeutic avenues in periodontal tissue engineering.

## 1. Introduction

Periodontal tissue constructs perform significant functions in the support of tooth structures under occlusal/masticatory loading conditions [1] and in defense against the invasion of various oral microorganisms [2,3]. In particular, the periodontal complex consists of tooth-supportive structures that are germane in remodeling, preservation, or maintenance of tissues including the cementum (the mineralized layer on the tooth root surface), periodontal ligament (PDL; fibrous connective tissue), and alveolar bone (force-responsive mineralized tissue) [4]. To optimize the physiological functionalities in dental and craniofacial complexes, periodontal complexes must show specific orientations of fibrous tissue bundles, collagenous fiber integration between mineralized surfaces, and spatiotemporal orchestration of compartmentalized tissue types [5,6,7].

Periodontitis, a highly prevalent inflammatory infectious disease, commonly induces tissue destruction of the periodontal complex in humans [3]. This disease is initiated by bacterial products such as lipopolysaccharide (LPS), which can stimulate cytokines to signal precursor cells to differentiate and activate osteoclastic cells and/or the periodontal inflammatory process by bacterial biofilm [8,9]. Periodontal disease or traumatic injury can lead to destruction of the periodontal complex including hard tissue resorption, destruction of the PDL, or biomechanical malfunctions due to the loosening of PDL anchorages from mineralized tissue surfaces and subsequent tooth loss [3]. Various non-surgical periodontal therapies have been still performed such as scaling, root planning, or anti-infective therapies to eliminate inflammation, periodontitis-causing bacteria, and microbial biofilm [9,10,11,12]. However, the periodontal surgery is critically required for periodontal health and highly expected to regenerate tooth-supportive structures and restore periodontal functions such as pocket reduction procedures and regeneration procedures (guided tissue regeneration; GTR or guided bone regeneration; GBR) [10,13]. In severe cases of periodontitis, dental prosthetics including dental implants or dentures are commonly required after tooth-extraction socket healing and bone regeneration; these approaches use various bioactive molecules [14,15], osteoconductive biomaterial fillings, or stem cell therapies [16,17,18,19] to regenerate implant-supportive bone tissues [20]. Many different types of dental implants have been developed and are widely utilized after alveolar bone regeneration treatments, such as bone regeneration for bone-implant anchorage to improve mechanical stability. 

**Figure 1:**
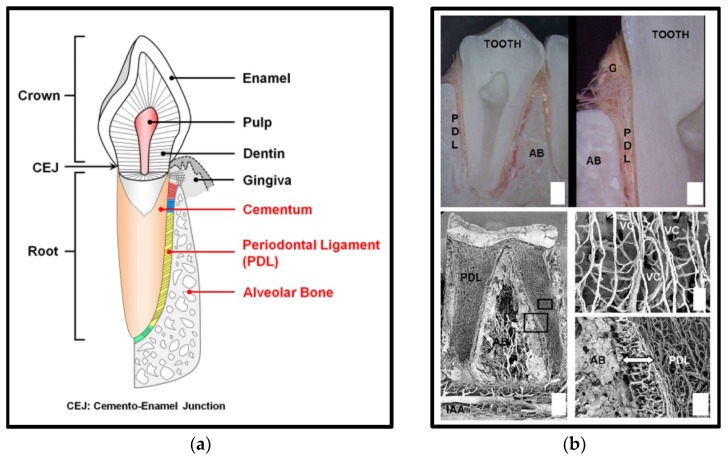
Anatomical schematic illustration of the periodontal tissue complex and the structure of periodontal tissues (**a**). (**b**) Periodontal ligament (PDL), alveolar bone (AB), gingiva (G), inferior alveolar artery (IAA), Volkmann’s canals (VC) [22,23].

Recently, periodontal complex (cementum-PDL-bone, Figure 1 regeneration has received significant attention as a way to maintain natural dental structures and re-functionalize healthy natural teeth in dental tissue engineering [4,21]. For tissue regeneration, different delivery systems have been developed for multiple tissue formations. However, spatiotemporal compartmentalization is a critical requirement for micron-scaled multiple tissue regeneration and functional restoration [22]. Recently, Park *et al.* investigated the freeze-casting method for longitudinal pore structures with controlled angulations (Figure S1) [22]. Directional ice-crystal formation and growth could be controlled by the freezing directions and would be longitudinal pore structures after being removed by the freeze-drying (Figure S1). The angulated pore platform had the structural similarity to oblique PDL interface, which is mostly numerous fibers and has important biomechanical functions to support tooth structures in five principal PDL fiber groups [22]. Therapeutic strategies for the regeneration of hierarchical architectures involved in systematic integrations for the re-establishment of tooth-supportive functions are currently limited mainly by the sub-micron-scaled interfaces and systematic compartmentalization to mimic periodontal structures and functions. Therefore, periodontal complex neogenesis is a formidable challenge in dental and craniofacial tissue engineering. Recently, periodontal regeneration strategies have been developed to facilitate spatiotemporal morphogenesis and the organization of fibrous connective tissue and mineralized structures in preclinical scenarios.

## 2. Multi-Phasic Biological Architecture for Periodontal Complex Formation

### 2.1. Periodontal Ligament (PDL) Cell Sheet Engineering Approaches for Ligamentous Tissue Formation

**Figure 2:**
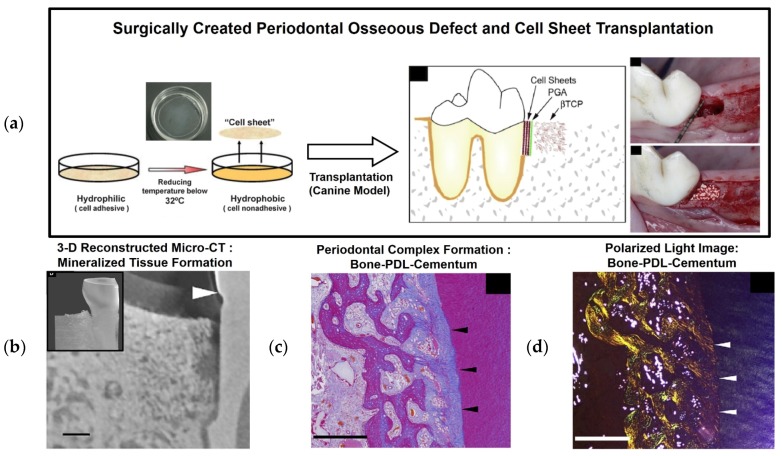
Surgical creation of a three-wall defect in a canine model. After cultivating and harvesting cell sheets, a PGA (poly-glycolic acid) membrane was placed on the tooth-root surface, and β-tricalcium phosphate (β-TCP) was used to fill in the defect (**a**) [26,27]. (**b**–**d**) Micro-computed tomography (Micro-CT) and histology were performed to assess bone regeneration and mineralized tissue formation on the root surface. Moreover, histological and polarized microscopic images demonstrated PDL regeneration with oblique orientations and periodontal attachment. The scale bars: 1 mm for (**b**) and 500 μm for (**c**,**d**) [27].

Cell sheet technology is a promising, clinically applicable approach for periodontal tissue regeneration [24,25,26,27]. Briefly, with this approach, the thermally responsive polymer poly(*N*-isoproplyacrylamide) (PIPAAm) is chemically grafted onto the cell culture plate surface, which is reversibly switched via temperature changes at 37 °C or below 32 °C for hydrophobic or hydrophilic surfaces, respectively [28,29]. This biomaterial-free, cell-rich approach has reported various successful clinical transplantations to wound sites for cardiac patches [30,31] and cornea reconstruction [28]. Recently, PDL stem cell sheets were transplanted for PDL and cementum tissue regenerations around the tooth root surface (Figure 2) [32,33,34,35]. Heterogeneous PDL cells can be cultivated under normal culture conditions (35–37 °C) and develop cell-cell and cell-matrix interactions on the PIPAAm-coated surface over time. To remove an individual cell sheet from the plate surface, the temperature-sensitive polymeric material can be made hydrophilic and undergo swelling below 32 °C (Figure 2). Multi-layered PDL cell sheets have been transplanted to periodontal defect sites with osteoconductive material (β-tricalcium phosphate; β-TCP) for cementum-PDL regeneration and bone formation, respectively (Figure 2) [27]. The results showed that a three-layered PDL cell sheet with an osteoconductive material approach could promote the regeneration of a clinically relevant multi-compartmentalized periodontal [27]. 

### 2.2. Proof-of-Concept: Multi-Phasic Integration with Cell Sheets and PCL Scaffolds

**Figure 3:**
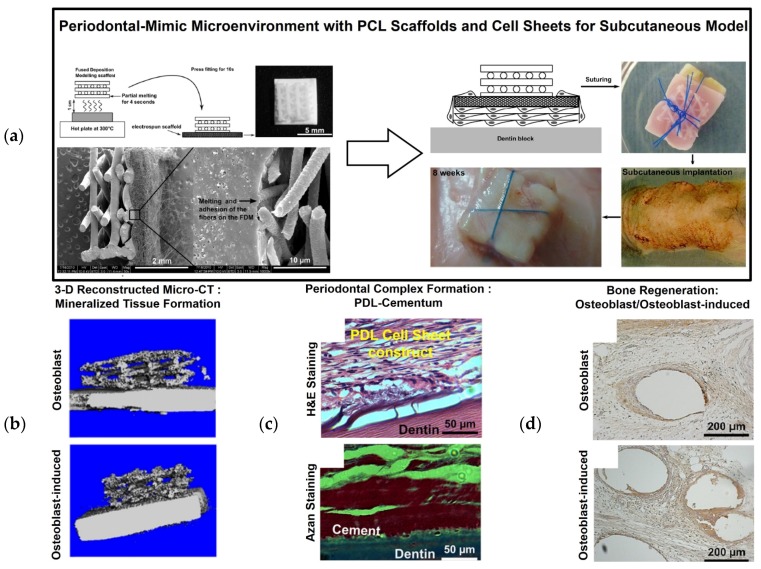
Periodontal regeneration construct using a poly-ε-caprolactone (PCL) nanofibrous scaffold with cell sheets. Biphasic scaffolds were fabricated using electrospinning and fused deposition modeling (FDM) (**a**). The cultured cell sheets were placed on top of the electrospun PCL membrane, and the human dentin blocks were assembled for subcutaneous transplant in athymic rats. Micro-CT demonstrated mineralized tissue formation with the dentin block (**b**), and hematoxylin and eosin (H&E) staining showed cementum and PDL regeneration on the dentin surface (**c**). Alkaline phosphatase staining showed bone-like tissue formation (**d**) [37].

Recently, a biphasic scaffold with PDL cell sheets was investigated using biodegradable poly-ε-caprolactone (PCL) nanofibrous scaffolds [36,37]. Briefly, the fused deposition modeling (FDM) technique was used to manufacture 20% hydroxyapatite (HA) mixed PCL scaffolds for bone tissue formation, and the electrospun PCL scaffolds were thermally incorporated for PDL regeneration (Figure 3). After assembling the human dentin block, PDL cell sheets, and biphasic scaffold, which was compartmentalized into the electrospun PCL scaffold and FDM HA/PCL scaffold, the constructs were implanted subcutaneously (Figure 3). The results showed that the electrospun PCL membrane facilitated the positional stability of PDL cell sheets on the dentin surface and promoted fibrous connective tissue attachment between the newly formed PDL and bone. Moreover, this highly porous interface significantly helped to prevent host cell infiltrations, which might unpredictably influence the PDL cell sheet constructs and regeneration of the PDL [37]. Therefore, the spatial compartmentalization of the periodontal-mimic construct enhanced the micron-scaled, hierarchical periodontal tissue neogenesis. Based on this proof-of-concept study, FDM scaffolds with chemical surface modification using calcium phosphate (CaP) may be used to improve osteoconductivity and enhance bone regeneration [36,38]. 

### 2.3. Preclinical Study: PDL Cell Sheet Transplantation with Electrospun CaP-PCL Scaffolds

**Figure 4:**
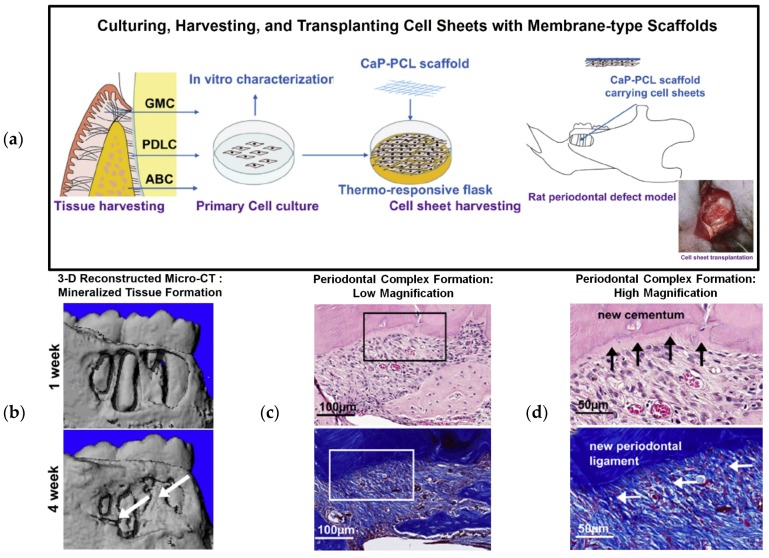
Cell sheet with CaP-PCL membrane transplantation for periodontal tissue regeneration and attachment. Schematic illustration of cell sheet harvesting and transplanting with a CaP-PCL membranous scaffold (**a**). 3-D reconstructed micro-CT images demonstrate bone regeneration at 1 and 4 weeks (**b**), and H&E staining showed periodontal tissue formation around the tooth surface at low and high magnifications (**c**,**d**). Cementum formation was analyzed using Azan staining, and PDL fibrous connective tissue attachment was analyzed by H&E staining [39].

Osteoconductively modified biphasic scaffolds using CaP with PCL nanofibrous scaffolds have been developed and assembled with cell sheet constructs for multiple periodontal tissue regenerations for periodontal fenestration defects [36,38]. To carry the cultivated cell sheets, electrospun PCL membrane was manufactured under the following conditions: 20 μL/h, 7 kV, and a tip-collector distance of 4 cm for 5 min. The electrospun nanofibrous PCL scaffolds were modified for hydrophilicity using NaOH and had CaP-coating onto the surface of the modified scaffolds. To cover the membranes, 15 w/v% PCL solution with chloroform/dimethylformamide (9.1 vol/vol) was electrospun onto a flat collector surface under the following conditions for 30 min: feed rate of 2 mL/h, 10 kV, and a tip-collector distance of 20 cm [39]. The limitation of the PDL cell sheet technique for periodontal tissue regeneration is its ability to position and secure cell sheets on the correct defect site of the tooth root surface. In addition to the ectopic, subcutaneous model in previous work, additional fabricated CaP-coated PCL membranes have facilitated delivering and securing biological cell sheets to the root-exposed periodontal defect [39]. Moreover, the osteoconductive characteristics of CaP-PCL membranes can facilitate osteogenic acceleration and the regeneration of tooth-supportive mineralized tissues [36], and PDL cell sheets can significantly compartmentalize PDL interfaces between the cementum and bone architecture [39]. Interestingly, the PDL cell sheets significantly promoted increased cementogenesis and bone formation for periodontal attachments compared to different cell sheet groups such as gingival margin-derived cells and alveolar bone cells (Figure 4). Due to their high flexibility, easy fabrication, and bioactive characteristics, this assembled construct of PDL cell sheets and modified PCL membranes has the potential for clinical periodontal regeneration [39].

## 3. CAD-Based Compartmentalized Designs for Periodontal Complex Formations

### 3.1. Proof-of-Concept Studies: the Engineered Periodontal-Mimic Microenvironment in the Subcutaneous Model

**Figure 5:**
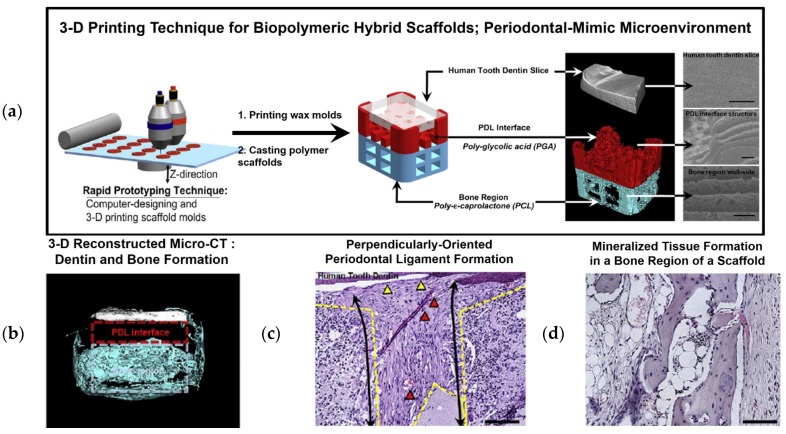
Proof-of-concept using a periodontal-mimic scaffolding system with a human dentin slice. (**a**) CAD-based 3-D wax molds were manufactured and cast with biodegradable polymeric materials, including poly-glycolic acid (PGA) for the PDL interface in red and poly-ε-caprolactone (PCL) for the bone region in blue. (**b**) Micro-CT and (**c**,**d**) histological image assessments. In the micro-CT image, mineralized tissue (blue) was spatially formed in a bone region of the hybrid scaffold without bone infiltration into the PDL interface (red dashed line). Histologically, dense fibrous connective tissues formed with blood vessels (red triangles) in a perpendicular orientation (black arrows), and cementum-like tissues underwent limited deposition on the dentin surface (yellow triangles). Bone region exhibited mineralized tissue formation. The scale bar: 50 μm [40].

Using computer-aided design (CAD) and 3-D printing technology, a hybrid scaffold was created with compartmentalized interfaces for the PDL and bone tissues, including PDL-mimetic architectures, which have perpendicular orientations to prepared human dentin slices [40]. A 3-D wax printer was utilized to manufacture the wax molds, and biopolymeric materials were cast with poly(glycolic acid) (PGA) in the PDL interface and poly-ε-caprolactone (PCL) in the bone architecture, which were selected for their rapid and slow biodegradation rates for PDL fibrous tissue and mineralized tissue formations, respectively. To mimic the periodontal microenvironment, human dentins were prepared with approximate dimensions of 3.0 mm × 4.0 mm × 0.8 mm and dentinal tubule exposures (Figure 5). Then, an ectopic periodontal tissue regeneration model was constructed in immunodeficient mice using an engineered periodontal microenvironment created to assemble a human dentin slice, PGA-cast PDL scaffold, and PCL-cast bone architecture [40]. This subcutaneous proof-of-concept study produced the interesting findings that (1) fibrous tissue was formed on the designed PGA structures with perpendicular or oblique orientations to the dentin slices; (2) mineralized tissues were formed in a bone construct and dentin surface, which could represent bone and cementum-like tissue formation for PDL functioning; and (3) ligament-bone tissues were generated with specific, highly controllable compartmentalization and organization. In particular, the most notable finding was the geometric or architectural promotion cues to precisely control connective tissue orientations in 250- to 300-μm interfaces with significant predictability. In addition to fibrous tissue orientation, the limited infiltrations of the regenerated bone tissue into the PDL architecture facilitated spatiotemporal tissue organization, even though the hybrid scaffolds were highly interconnected [40]. 

### 3.2. Preclinical Study: Image-Based Scaffolds for Periodontal Complex Regeneration 

**Figure 6:**
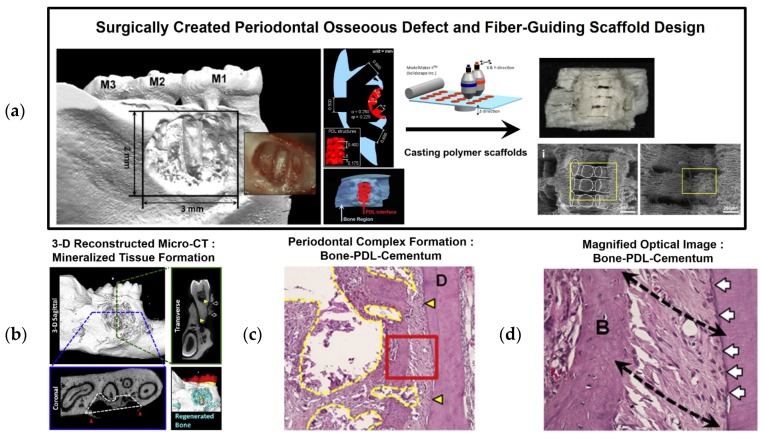
Preclinical study using image-based 3-D printing technology for periodontal complex regeneration. (**a**) After surgically creating the periodontal fenestration defect on the buccal side of a mandible, customized fiber-guiding scaffolds were designed and manufactured using 3-D printed wax molds. (**b**) 3-D reconstructed micro-CT and (**c**,**d**) histological image assessments for bone-PDL-cementum. D: dentin; B: bone; yellow triangles: surgically created defect regions on the dentin surface; black dashed arrows: fibrous tissue orientation; white arrows: calcified layer deposition on the dentin surface [6].

As a customized scaffolding approach, Park *et al.* developed a computed tomographic (CT) image-based fiber-guiding scaffold, which includes compartmentalized architecture for PDL and bone formation in a single system. Interior and exterior designs in a fiber-guiding scaffold constituted tissue regeneration platforms based on periodontal-mimic hybrid scaffolds in the proof-of-concept study [6,41]. Using CT-scanning and 3-D reconstructed image datasets of periodontal defects, fiber-guiding scaffolds were created using a CAD-based design tool. The designed fiber-guiding scaffold has multiple levels of compartmentalization, including a PDL interface with perpendicularly oriented architecture to the tooth-root surface topography and a bone region with open structures with porosity to allow tissue infiltration (Figure 6) [6]. Using a 3-D wax printer, wax molds were manufactured, and biocompatible PCL biopolymers were cast into the fabricated molds (Figure 6). As a result, the implanted fiber-guiding scaffolds guided the regeneration of tooth-supportive structures, and bone tissue formation proceeded into the bone region of the fiber-guiding scaffold and PDL orientation, with cementogenesis occurring on the tooth root surface. In addition to multiple tissue formation and fibrous connective tissue angulation, regenerated ligamentous tissues were integrated with the mineralized tissue surfaces, and the complex structures provided tooth-supportive functioning restoration [6,40]. Compared to fiber-guiding scaffolds, conventional salt-leached PCL scaffolds with random porous internal architectures provided unpredictable tissue compartmentalization and uncontrollable fibrous tissue orientations [6,41].

## 4. Clinical Case Study Using 3-D Printing Technology

**Figure 7:**
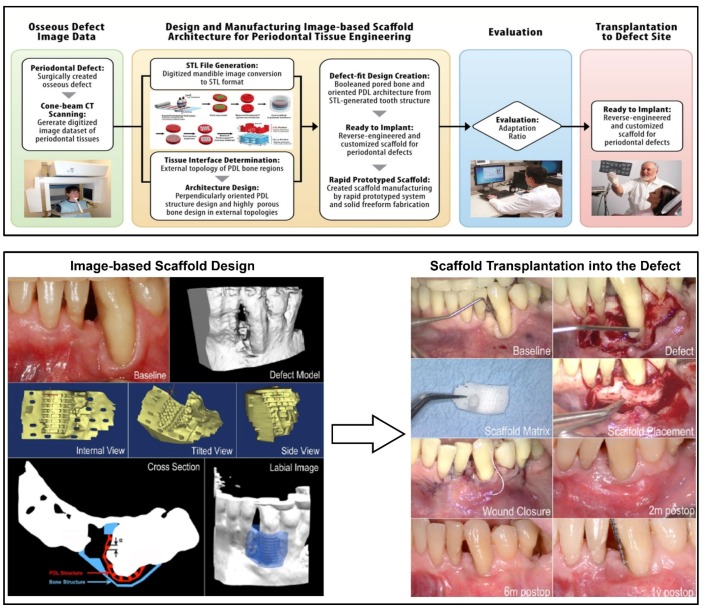
Flow chart for the design of customized, fiber-guiding scaffolds and clinical transplant of the 3-D printed, fiber-guiding scaffold to a labial defect. After the computer design of the customized scaffold, a 3-D printer was used to manufacture the PCL scaffolds. After pre-operative treatments, the PCL scaffold was placed at the defect with poly-D and L-lactic acid pins to secure the scaffold. The implanted site showed no signs of inflammation or infection during the first year after treatment [41,42].

Recently, a periodontal defect-specific, customized 3-D scaffold was clinically transplanted into a large labial periodontal defect [42]. The first report using 3-D printing technology for periodontal tissue regeneration demonstrated that this technology represents a promising approach to the design and manufacture of complicated, unpredictable geometries. After scanning the patient defect by cone-beam CT, 3-D reconstructed image datasets were utilized to design fiber-guiding scaffolds (Figure 7), and a 3-D printer was used to manufacture bioresorbable, PCL scaffolds with high adaptability (adaptation ratio: 82% ± 7%) [42]. 

The fiber-guiding scaffold covered the entire defect region and was positioned securely using biodegradable screws. The transplantation region showed no signs of chronic inflammation, infectious symptoms, or dehiscence for 12 months. However, the scaffold was slightly exposed at the 13th month and was removed at the 14th month due to a large dehiscence around the scaffold exposure [42]. The retrieved scaffold was fixed and histologically evaluated for tissue ingrowth, attachment, and inflammation and assessed for scaffold degradation using gel permeation chromatography (GPC). The primary clinical findings showed connective tissue healing and limited bone repair. The change in mean molecular mass of the PCL was 24.1% for 14 months compared to pre-implanted PCL scaffolds with an approximate 90-kDa molecular weight [42]. Although the first clinical trial for periodontal repair showed unexpected results in the long-term period, the prospect of using rapidly biodegradable polymeric materials and higher-resolution 3-D printing technology will likely improve the current limitations of personalized dentistry and medicine [42]. 

## 5. Conclusions

Various techniques and approaches have been developed for periodontal tooth-supportive complex regeneration with functioning restoration. Herein, we reviewed the recent technological advances in this field, including compartmentalized designs at micron-scale geometries with multiple tissue regeneration, integration and periodontal attachments. In future work, architectural designs and tissue-engineered implants for periodontia must address key considerations: (1) more controllable and predictable geometries for fibrous connective tissue angulations; (2) topographical approaches to optimize cementum and bone regeneration; and (3) appropriate Food and Drug Administration (FDA)-approvable biomaterials for rapid degradation. In particular, periodontal attachment may be the key to preserving and supporting natural teeth instead of dental prosthetic implants. Furthermore, these promising advances will likely provide the opportunity for huge paradigm shifts in dental tissue engineering, from replacement to regeneration. 

## Supplementary Materials

The following are available online at www.mdpi.com/1996-1944/9/1/57/s1.
