# Supplementary material for: Advanced Engineering Strategies for Periodontal Complex Regeneration
Source: Materials (Basel). 2016 Jan 18;9(1):57. doi: 10.3390/ma9010057 (PMC5456552; doi:10.3390/ma9010057)
Supplement: Supplementary file 1 [file materials-09-00057-s001.pdf]

Supplementary Materials: Advanced Engineering Strategies for Periodontal Complex Regeneration

Chan Ho Park, Kyoung-Hwa Kim, Yong-Moo Lee and Yang-Jo Seol

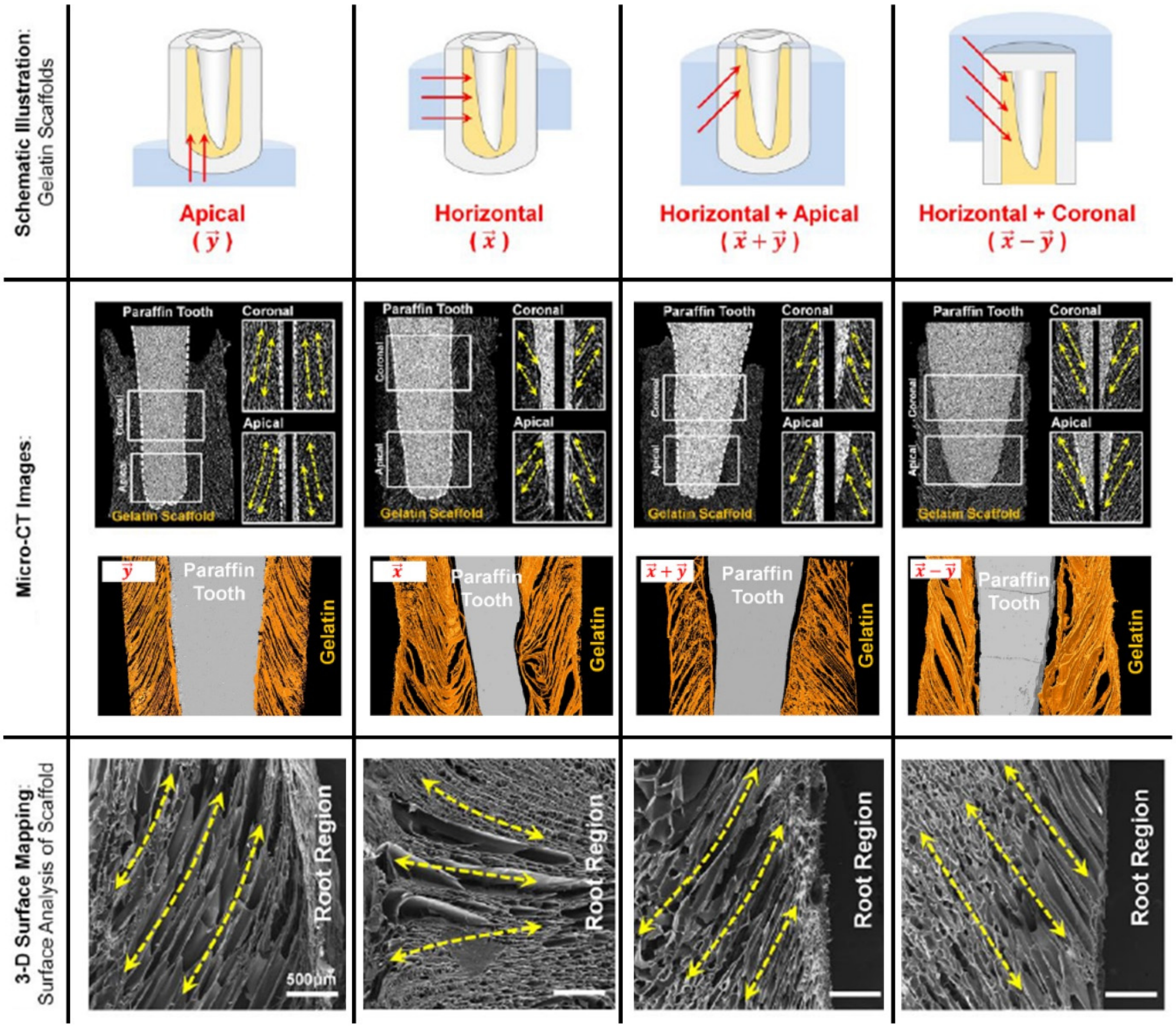

Figure S1. The freeze-casting method to create longitudinal pore structures with specific angulations. (**Top**) The schematic illustration to show different freezing directions can create and control periodontal ligament PDL-mimic architectures in gelatin scaffolds. (**Middle**) Digitized cross-sectional views and 3-D reconstructed images by micro-computed tomography (Micro-computed tomography (Micro-CT)) showed the directional pore structures in gelatin scaffolds (orange-colored). (**Bottom**) Scanning electron microscopic (SEM) images provided the longitudinal pore structures by the freeze-casting method. Yellow dashed arrow-lines represent the pore directions. Scale bar: 500 µm.
